# 고위험 임부의 태교실천, 자존감 및 사회적 지지가 모아애착에 영향을 미치는가?: 횡단적 조사 연구

**DOI:** 10.4069/kjwhn.2022.12.16

**Published:** 2022-12-29

**Authors:** Da-In Kang, Euna Park

**Affiliations:** Department of Nursing, Pukyong National University, Busan, Korea; 부경대학교 간호학과

**Keywords:** High-risk pregnancy, Maternal-fetal relations, Self concept, Social support, 고위험 임신, 태아애착, 지존감, 사회적 지지

## Abstract

**Purpose:**

The incidence of high-risk pregnancies is increasing in Korea as the birth age increases due to late marriage. Maternal-fetal attachment is an important factor that affects children even after childbirth, but it is difficult for high-risk pregnant women to form maternal-fetal attachment. The current study aimed to explore whether *taegyo* practice (i.e., pregnant women’s efforts for fetal good growth and development), self-esteem, and social support influenced the degree of maternal-fetal attachment in women with high-risk pregnancies.

**Methods:**

The participants included 226 pregnant Korean women at ≥20 gestational weeks, hospitalized with 15 high-risk pregnancy conditions as defined by the Ministry of Health and Welfare. Recruitment via convenience sampling was done at four sites in Busan, Korea. Surveys were distributed and collected from February 1 to 28, 2022. Data analysis was conducted using descriptive statistics, the t-test, one-factor analysis of variance, Pearson correlation coefficients, and hierarchical multiple regression.

**Results:**

On average, participants were 33.97±4.23 years of age and at 31.65±6.23 gestational weeks. Preterm labor (35.4%) and gestational diabetes (21.0%) were the most common high-risk conditions. Maternal-fetal attachment was positively correlated with *taegyo* practice (r=.70, *p*<.001), self-esteem (r=.53, *p*<.001), and social support (r=.53, *p*<.001), all with statistical significance. Taegyo practice (β=.50, *p*<.001) and social support (β=.17, *p*=.030) explained 53% of variance in maternal-fetal attachment in women with high-risk pregnancies.

**Conclusion:**

Nurses caring for women with high-risk pregnancies during hospitalization can use these findings by promoting *taegyo* practice and enhancing social support to increase maternal-fetal attachment.

## Introduction

여성의 인생에서 임신과 출산은 중요한 사건이며, 임신과 출산 시기는 임부에게 신체적, 정신적, 사회적으로 가장 변화가 심한 시기이다. 특히 정상 임신이 아닌 고위험 임신인 경우에는 임부나 태아의 건강과 생명이 위협받을 수 있고[1] 신체적, 정신적 긴장으로 여러가지 정서적인 문제가 초래될 수 있으므로[2] 고위험 임신에 대한 관리는 중요한 사회적 문제로 대두되고 있다.

2021년 초산모의 평균 출산 연령은 33.4세로, 1990년 25.9세, 2010년 30.1세와 비교하여 지속적으로 증가하고 있으며, 이는 인구 1,000명 당 출생아 수를 나타내는 가임기 여성의 연령별 출산율에서 30–34세 76.1명, 35–39세 43.5명으로 30대의 출산율이 가장 높게 나타난 것을 통해서도 확인할 수 있다[3]. 이와 같은 연령별 출산율의 변화로 난임과 관련된 시술의 발달과 함께 보조생식술을 이용한 임신이 늘어나게 되면서 다태아 임신 및 고위험 임신의 발생이 증가하는 경향을 보이고 있다[4]. 고위험 임신을 진단받은 임부의 수는 2009년 27,223명에서 2020년 139,476명으로 약 10년 동안 5배 이상 급증하였다[5]. 이와 관련하여 2015년, 정부에서 3대 고위험 임부를 대상으로 시작한 의료비 지원사업이 점차 확대되어 2019년 하반기부터는 조기진통, 분만 관련 출혈, 중증 임신중독증, 양막 조기파열 등의 19대 고위험 임신 질환을 대상으로 지원하고 있으나[6], 병원에서는 모아애착을 향상시킬 수 있는 출산 교실 운영 등과 같은 대부분의 프로그램을 정상 임부에 초점을 두고 실시하는 경우가 많아 고위험 임부에 대한 관심이 부족한 실정이다.

모아애착은 출산 전 모아관계의 출발점으로 임부와 태아가 정서적인 유대감을 가지는 행위이다[7]. 고위험 임부는 정상 임부에 비해 임신 기간 동안 태아와 자신의 안녕을 예측하기 힘든 상태이므로 걱정·불안과 같은 부정적인 정서를 더 많이 경험하게 되며[8], 이로 인해 모아애착 형성에 어려움을 겪는 것으로 나타났다[9]. 임신 중 모아애착의 형성은 출산 후 자녀와의 관계에 영향을 미치며[10], 모체의 모아애착이 낮은 경우 영아는 까다로운 성격을 가지는 경우가 많고, 수면과 적응 행동 발달에도 문제가 발생한다고 보고한 체계적 문헌고찰[11]을 고려하면 자녀의 심리적, 신체적인 발달에도 영향을 미치는 요소가 될 수 있으므로 그 중요성이 매우 높다.

임부의 모아애착에 영향을 미치는 요인 중 하나로 태교실천이 포함된다[12]. 태교실천이란 태아를 인식하고 최적의 성장발달을 달성하기 위한 출산까지의 임부의 노력을 의미한다[13]. 우리나라에서 태교실천은 임부가 마음을 안정하고 출산을 기다리며 정성을 다하는 중요한 과정으로 여기고 있다[14]. 태교실천은 태아에 대한 존중과 사랑에서부터 시작되며[15], 올바른 태교를 실천하기 위해서는 양질의 임신 생활을 통한 임부 자신의 행복이 바탕이 되어야 한다[2]. 하지만 고위험 임부는 장기간의 입원 생활이나 치료와 관련된 활동 제한 등으로 자신의 임신 과정에 대해 전반적으로 행복감을 가지기 어려울 수 있고[16], 현재 상태에 대한 불확실성이 높아[17] 태교실천 정도에 영향을 주어 태아와의 애착 형성에 저해요소로 작용할 수 있다[12].

자존감은 자신을 가치 있는 사람이라고 생각하는 정도로[18], 태아와의 애착 형성[19,20] 및 어머니로서의 역할 인식에[19] 중요한 요인이 된다. 임부의 자존감이 높을수록 임신과 출산의 어려움을 더 잘 극복하는 것으로 보고되었다[21]. 그러나 고위험 임부를 대상으로 자존감 정도를 확인한 국내 연구는 미비한 상태이다.

임부에 대한 사회적 지지는 자존감을 높여주는 역할과 함께 임신 및 분만과 관련된 충격을 완화하는 요인이 될 수 있다[12]. 사회적 지지는 스트레스 상황으로 인해 발생하는 부정적인 영향을 완화시켜 주는 행위로, 개인이 대인관계를 통해 얻을 수 있는 긍정적 자원이다[22]. 사회적 지지가 높을수록 임부의 불안이 감소되고[23], 신체적, 정신적인 안녕감이 높아지며[22], 조산 발생률이 낮아진다[24]. 또한 태아와의 애착이 향상될 수 있으며[25], 모아애착의 강도와 빈도 및 질에 영향을 미친다는[26] 측면에서 볼 때 고위험 임부에 대한 사회적 지지는 그 요구도가 더 높을 수 있다.

지금까지 모아애착에 영향을 미치는 요인과 관련된 선행 연구는 주로 정상 임부에만 초점을 맞추고 있으며, 임부의 태교실천 정도가 높을수록[10], 사회적 지지가 높을수록[27], 자존감이 높을수록[20] 모아애착의 정도가 높은 것으로 나타났다. 국내 고위험 임부의 모아애착에 관한 연구는 조기진통 임부[28], 불임 치료 임부[29], 유산을 경험한 임부[30], 임신성 당뇨병 임부[31] 등과 같이 일부 고위험 임부에 집중되어 있고, 국가에서 지정한 다양한 고위험 임신 질환을 진단받은 임부를 대상으로 한 연구는 미비한 실정이다.

이에 본 연구에서는 고위험 임부가 임신 과정에서 겪게 되는 모아애착, 태교실천, 자존감, 사회적 지지 정도와 이들 간의 관계를 규명하고 모아애착에 영향을 주는 요인을 파악함으로써, 고위험 임부를 위한 체계적이고 효과적인 교육 프로그램의 방향 제시와 간호 중재 개발의 이론적 근거가 되는 기초자료를 제공하고자 하였다.

## Methods

Ethics statement: This study was approved by the Institutional Review Board of Pukyong National University (1041386-202201-HR-4-02). Informed consent was obtained from the participants.

### 연구 설계

본 연구는 고위험 임신을 진단받은 임부의 모아애착 및 태교실천, 자존감, 사회적 지지 정도와 이들 변수 간의 관계를 파악하고, 모아애착에 영향을 주는 요인을 확인하기 위한 횡단적 조사연구이다. 본 연구는 STROBE 보고지침[32]에 따라 기술하였다.

### 연구 대상

본 연구 대상자는 부산에 소재한 1개의 2차 의료기관과 3개의 1차 의료기관인 여성병원을 포함한 총 4개 병원에 입원하고 있는 고위험 임부를 대상으로 하였다. 고위험 임신을 진단받은 임부를 대상으로 본 연구의 목적을 이해하고 자발적인 참여로 연구에 동의한 자로 한정하여 편의 표집하였다. 구체적인 선정기준은 15대 고위험 임신 질환[6]을 진단받고 입원한 임부, 임신 20주 이상의 임부, 연구의 목적을 이해하고 자발적으로 참여에 동의한 임부이며, 초기에 국가에서 지정한 고위험 임신 질환이 모두 포함되도록 계획하였으나 의료진의 판단 하에 상태가 위중하고 응급 분만이 예상된다고 판단된 양수과다증, 신질환, 태반조기박리와 산욕기 관련 진단명인 분만관련 출혈 등의 4대 고위험 임신질환은 제외하여 다음과 같은 15대 고위험 임신을 진단받은 임부를 대상으로 하였다: 조기진통, 임신성 당뇨병, 양막의 조기파열, 다태 임신, 임신과 구토, 전치태반, 자궁 경부 무력증, 고혈압, 절박유산, 자궁 및 자궁의 부속기 질환, 분만 전 출혈, 양수과소증, 중증 임신중독증, 자궁 내 성장제한, 심부전. 제외기준은 자가보고에 따라 정신질환을 진단받거나 향정신성 약물을 복용하고 있는 임부, 의사소통에 어려움이 있는 임부, 의료진의 판단 하에 상태가 위중하거나 응급 분만이 예상되는 고위험 임부로 하였다. 연구 대상자 수는 G-power 3.1.9.7 program을 이용하였고, 효과 크기는 선행 연구[30]에 근거하여 .15 (중간 크기), 유의수준 α=.05, 검정력 .80으로 예측변수 14개(일반적 특성 6개, 산과적 특성 5개 및 태교실천, 자존감, 사회적 지지)를 투입하였을 때 필요한 대상자 수는 194명이었다. 탈락률 15%를 고려하여 총 230명을 대상으로 자료를 수집하였고, 누락된 문항이 있거나 불성실한 응답을 한 설문지 4부를 제외한 226명의 설문지를 최종 분석에 사용하였다.

### 연구 도구

#### 모아애착

본 연구에서 모아애착은 Cranley [33]가 개발한 모아애착도구(Maternal-Fetal Attachment Scale)를 Kim [34]이 수정·번안한 도구를 승인을 얻은 후 사용하였다. 본 도구는 총 24개 문항으로 자신과 태아의 구별 3문항, 태아와의 상호작용 5문항, 역할 수용 4문항, 태아의 특성과 의도에 대한 추측 6문항, 자기 헌신 6문항으로 구성되어 있다. 도구의 척도는 4점 Likert 척도이며(‘전혀 안 했다’ 1점, ‘항상 그랬다’ 4점), 점수 범위는 24–96점으로 점수가 높을수록 모아애착 정도가 높음을 의미한다. 개발 당시 도구 신뢰도 Cronbach’s α는 .85, Kim [34]의 연구에서는 .89, 본 연구에서는 .96이었다.

#### 태교실천

본 연구에서 태교실천은 Choi와 Kim [35]이 임부를 대상으로 개발한 태교실천 도구를 Kim [34]이 수정·보완한 도구를 승인을 얻은 후 사용하였다. 본 도구는 총 37문항으로 권장행위 19문항과 금기행위 18문항으로 구성되어 있다. 도구의 척도는 5점 Likert 척도이며(태교 권장행위를 ‘전혀 하지 않는다’ 1점, ‘항상 한다’ 5점; 태교 금기행위를 ‘전혀 하지 않는다’ 1점, ‘항상 금한다’ 5점), 점수 범위는 37–185점으로 점수가 높을수록 태교실천 정도가 높은 것을 의미한다. 금기행위를 묻는 질문 중에는 개인에 따라서 임신 기간 동안에 접할 기회가 없는 행위들이 있을 수 있으므로 TV, 라디오, 비디오 등을 통한 간접 경험이나 만약 그러한 상황을 접할 경우를 가정하여 응답하도록 하였다. 개발 당시 도구 신뢰도 Cronbach’s α는 .96, Kim의 [34] 연구에서는 .89였고, 본 연구에서는 .90이었다.

#### 자존감

본 연구에서 자존감은 Rosenberg [36]가 개발한 자존감 척도(Rosenberg Self-Esteem Scale)를 Lee와 Won [37]이 번안한 도구를 승인을 얻은 후 사용하였다. 본 도구는 총 10문항으로 긍정적인 5문항, 부정적인 5문항으로 구성되어 있으며 3, 5, 8, 9, 10번은 역채점 문항이다. 도구의 척도는 5점 Likert 척도이며(‘전혀 그렇지 않다’ 1점, ‘매우 그렇다’ 5점), 점수 범위는 10–50점으로 점수가 높을수록 자존감 수준이 높음을 의미한다. 개발 당시 도구 신뢰도 Cronbach’s α는 .93, Lee와 Won [37]의 연구에서는 .89이었고, 본 연구에서는 .83이었다.

#### 사회적 지지

본 연구에서 사회적 지지는 Park [38]이 40문항으로 개발한 사회적 지지 척도를 Song [39]이 25문항으로 수정하였고, 이를 Lee와 Park [40]이 임부를 대상으로 수정한 도구를 승인을 얻은 후 사용하였다. 본 도구는 총 25문항으로 정서적 지지 8문항, 정보적 지지 5문항, 물질적 지지 6문항, 평가적 지지 6문항으로 구성되어 있다. 도구의 척도는 Likert 5점 척도이며(‘모두 그렇지 않다’ 1점, ‘모두 그렇다’ 5점), 점수 범위는 25점–125점으로 점수가 높을수록 산전 사회적 지지가 높음을 의미한다. 개발 당시 도구 신뢰도 Cronbach’s α는 .94, Song [39]의 연구에서는 .95, Lee와 Park [40]의 연구에서는 .96이었고, 본 연구에서는 .99였다.

#### 일반적 특성 및 산과적 특성

임부의 일반적 특성은 연령, 교육 정도, 종교, 직업, 결혼 만족도, 스스로 느끼는 건강상태 등의 6문항으로 구성되어 있다. 임부의 산과적 특성은 분만 예정일과 임신 주수, 계획 임신 여부, 임신 방법, 임신 횟수, 해당하는 고위험 임신 분류 등의 총 5문항으로 구성되어 있다.

### 자료 수집

본 연구는 2022년 2월 1일부터 2월 28일까지 부산에 소재한 1개의 종합병원과 3개의 여성병원의 병원 진료과 및 간호부를 방문하여 본 연구의 목적 및 참여 방법을 설명하고 자료 수집 승인을 얻은 후 시행하였다. 자료 수집 시 연구자가 해당 병원의 분만실 및 병동을 방문하여 모집 공고문을 게시하였고, 연구에 관심 있는 임부들에게 연구의 목적과 내용에 대해 설명한 후 연구 참여에 동의한 대상자에게 서면동의서를 받고 자기 보고식 설문지를 배부한 후 밀봉 봉투로 즉시 회수하였다. 연구자의 전화번호를 기재하여 설문지에 대한 의문이 있는 경우 연락할 수 있도록 하였다. 설문지의 작성 시간은 약 15–20분 정도 소요되었고, 설문지 회수 시 대상자에게 소정의 선물을 지급하였다.

### 자료 분석 방법

본 연구에서 수집된 자료는 IBM SPSS Statistics for Windows ver. 25.0 (IBM Corp., Armonk, NY, USA)을 이용하여 분석하였다.

고위험 임부의 특성을 알아보기 위해 빈도와 백분율, 평균, 표준편차의 기술통계를 실시하였다. 고위험 임부의 태교실천, 자존감, 사회적 지지 및 모아애착의 정도를 파악하기 위해 평균과 표준편차로 분석하였고, 고위험 임부의 특성에 따른 모아애착의 차이는 독립표본 t검정, 일원분산분석, 사후 검정 분석은 Scheffé test로 분석하였다. 고위험 임부의 태교실천, 자존감, 사회적 지지 및 모아애착 간의 상관관계를 파악하기 위하여 상관관계 분석을 하였으며, 고위험 임부의 모아애착에 영향을 미치는 요인을 확인하기 위해서 위계적 다중회귀분석을 하였다.

## Results

### 고위험 임부의 일반적 특성 및 산과적 특성에 따른 모아애착 차이

고위험 임부의 연령은 35세 미만이 127명(56.2%), 35세 이상이 99명(43.8%)이었으며, 평균 33.97±4.23세로 나타났다. 학력은 대졸이 166명(73.5%), 종교는 ‘무’인 경우가 144명(63.7%), 직업은 ‘유’인 경우가 144명(63.7%), 결혼 만족도는 만족하는 경우가 183명(81.0%), 건강상태는 보통이 136명(60.2%)이었다.

고위험 임부의 평균 임신 주수는 31.65±6.23주로 29주 이상이 148명(65.5%)이었다. 계획 임신 여부는 ‘예’인 경우가 146명(64.6%), 임신 방법은 자연임신이 159명(70.4%), 고위험 임부의 임신 횟수는 첫 번째가 144명(63.7%)으로 가장 많았다. 고위험 임부의 진단명은 복수 응답이었으며, 조기진통 125명(35.4%), 임신성 당뇨병 74명(21.0%), 양막의 조기파열 20명(5.7%), 다태 임신 19명(5.4%), 임신과 구토 18명(5.1%), 전치태반 17명(4.8%), 자궁 경부 무력증 14명(4.0%), 고혈압 14명(4.0%), 절박유산 12명(3.4%), 자궁 및 자궁의 부속기 질환 11명(3.1%), 분만 전 출혈 11명(3.1%), 양수과소증 7명(2.0%), 중증 임신중독증 4명(1.1%), 자궁 내 성장 제한 4명(1.1%), 심부전 3명(0.8%) 순으로 많았다(Table 1).

본 연구에서 고위험 임부의 모아애착은 결혼만족도(F=31.78, *p*<.001), 건강상태(F=12.61, *p*<.001), 계획 임신 여부(t=2.98, *p*=.003)에서 유의한 차이가 있었고 계획 임신 여부는 있는 경우가 없는 경우보다 모아애착이 높았다. 이를 Scheffé test로 사후검증을 실시한 결과에서 결혼만족도는 ‘만족’이 ‘보통’과 ‘불만족’인 경우보다(F=31.78, *p*<.001), 건강상태는 ‘좋음’과 ‘보통’이 ‘나쁨’보다(F=12.61, *p*<.001) 태아 애착이 높았다(Table 1).

### 고위험 임부의 모아애착과 태교실천, 자존감, 사회적 지지의 정도

본 연구에서 고위험 임부의 모아애착은 76.64±14.82으로 중등도 이상 수준이었다. 태교실천 136.47±15.18점, 자존감 35.50±5.90점으로 중등도 이상 수준이었으며, 사회적 지지는 102.61±22.94점으로 높은 편이었다(Table 2).

### 고위험 임부의 모아애착, 태교실천, 자존감, 사회적 지지 간의 상관관계

본 연구에서 고위험 임부의 모아애착은 태교실천(r=.70, *p*<.001), 자존감(r=.53, *p*<.001), 사회적 지지(r=.53, *p*<.001) 모두와 통계적으로 유의한 정적 상관관계가 있는 것으로 나타났다(Table 3).

### 고위험 임부의 모아애착에 대한 영향요인

고위험 임부의 모아애착에 영향을 미치는 요인을 알아보기 위하여 위계적 다중회귀분석을 실시하였다. 회귀분석을 실시하기 위한 조건을 확보하기 위해 종속변수의 자기 상관과 독립변수 간의 다중공선성을 Dubin-Watson 지수와 분산팽창지수(variance inflation factor, VIF)를 이용하여 검토했다. 그 결과 Dubin-Watson 지수는 1.726으로 2에 가까워 자기상관이 없었으며, VIF는 1.076–2.743으로 10 이하이므로 독립변수 간의 다중공선성에 문제가 없었다.

모델 1에는 모아애착에 유의한 차이를 보인 일반적 특성 중 결혼 만족도와 건강상태, 산과적 특성 중 계획 임신을 투입하였고, 모델 2에는 모아애착에 유의한 상관관계를 보인 태교실천, 자존감, 사회적 지지를 투입하여 모아애착에 미치는 영향을 파악하였다. 이중 결혼 만족도, 건강상태, 계획 임신은 가변수(dummy variables)로 처리하여 회귀분석을 실시하였다. 모델 1의 설명력은 24%였고, 통계적으로 유의하였다(F=14.90, *p*<.001). 결혼만족도가 보통(β=–.36, *p*<.001)이거나 불만족(β=–.17, *p*=.005)인 경우 만족하는 경우보다 모아애착이 낮았으며, 건강상태가 ‘나쁨’이 ‘좋음’보다 모아애착이 낮았다(β=–.19, *p*=.016).

모델 2의 설명력은 53%로 모델 1에 비해 29% 증가하였으며, 통계적으로 유의하였다(F=46.28, *p*<.001). 태교실천(β=.50, *p*<.001)과 사회적 지지(β=.17, *p*=.030)가 높을수록 모아애착이 높은 것으로 나타났다(Table 4).

## Discussion

본 연구에서 고위험 임부의 모아애착에 가장 큰 영향을 미치는 변수는 태교실천이었다. 이는 정상 임부를 대상으로 모아애착에 영향을 미치는 요인을 분석한 연구[12]에서 태교실천이 가장 큰 영향을 미치는 변수로 나타난 것과 일치하는 결과로, 태교실천 관련 과학적, 실증적 연구를 지속적으로 수행하여 태교실천의 필요성을 확보하여야 할 것이다. 또한 의료인은 고위험 임신 질환에 대한 관리와 함께 태교실천의 중요성을 강조하고, 태교실천을 고위험 임부의 건강관리체계 내로 통합하는 방법을 모색할 필요가 있다. 현재 태교실천 프로그램은 주로 정상 임부를 대상으로 체험 중심 산전 프로그램[41], 태교 운동 프로그램[42] 등과 같이 특정 장소에서 활동적으로 수업에 참여하는 형태이다. 고위험 임부는 시간과 공간의 제약을 받는 경우가 많으므로, 여러 가지 교육매체 중 접근성과 편의성이 뛰어난 스마트폰을 통해 침상에서 유튜브나 어플리케이션 등을 활용한 자기주도 학습 프로그램을 개발하는 것이 필요할 것이다.

두 번째로 모아애착에 영향을 미치는 요인은 사회적 지지인 것으로 나타났다. 높은 사회적 지지를 경험하는 임부는 모아애착 행위를 더 많이 하게 되나[43], 사회적 지지가 낮은 임부는 모아애착의 질이 낮고, 태아에 대한 생각을 적게 할 가능성이 있다[26]. 배우자를 포함한 가족, 사회, 의료인은 고위험 임부에 대한 이해를 기반으로 임신 과정에 함께 참여하도록 적극적으로 격려하고 고위험 임부가 접근할 수 있는 다양한 사회적 지지체계를 마련하는 것이 모아애착을 높이는 데 효과적일 것으로 생각된다. 예를 들어 입원 기간 중 의료인의 고위험 임부에 대한 체계적인 정보 제공과 함께 고위험 임부들이 서로 비슷한 경험을 공유하고 교류할 수 있는 인터넷 기반 모임이 만들어진다면 또 다른 사회적 지지 체계가 될 수 있을 것이다. 고위험 임부가 신체적, 정신적으로 건강하게 출산을 준비하며 모아애착을 증진할 수 있도록 가족, 사회 및 의료인을 포함한 포괄적인 연계를 통해 함께 노력하는 것이 필요할 것이다.

본 연구에서 고위험 임부의 모아애착은 평균 76.64점으로, 정상 임부를 대상으로 한 선행 연구[44]에서의 평균 83.28점보다 다소 낮은 수준이었다. 이는 고위험 임신의 경우 임신의 진행이나 결과의 불확실성으로 인해 태아에 대한 애착 형성을 주저하는 경우가 많고[17], 장기간 입원이 필요한 고위험 임부는 신체적인 불편감과 스트레스가 높아져 모아애착이 감소한 측면이 있을 수 있다[28]. 특히 입원한 고위험 임부의 스트레스와 관련하여 본 연구 기간 중 전세계적 coronavirus disease 2019 (COVID-19)의 유행으로 임부들이 감염 위험에 대한 우려와 함께 감염병 예방과 보호에 대한 지식과 조치, 심리상담 등의 요구도가 높아진 점[45]을 고려한다면 외부적, 환경적 요인과 관련된 고위험 임부의 모아애착의 변화에 대한 추가적인 후속 연구가 필요할 것으로 생각한다.

또한 중등도 이상으로 확인된 태교실천(평균 136.47점)은 정상 임부를 대상으로 한 선행 연구[12]의 평균 138.74와 유사한 수준이었다. 그러나 고위험 임부의 경우 정상 임부보다 임신 유지 및 경과 관찰을 위해 침상 안정을 해야 하는 경우가 많아 태교보다는 TV 시청이나 인터넷 사용 등에 주로 집중하게 되어[16] 태교실천의 정도가 더 낮아질 가능성이 있으므로, 침상 안정 상태의 고위험 임부를 주대상자로 하는 태교실천 프로그램 마련이 필요할 것이다.

본 연구에서의 자존감은 평균 35.50점으로, 동일한 도구를 사용한 정상 임부 대상 국내 연구[20]에서의 37.15과 유사한 수준이나, 이란에서 정상 임부를 대상으로 한 연구[46]에서의 환산 점수인 42.65점보다는 다소 낮은 수준이었다. 이러한 차이는 국가 간의 문화적 차이와 관련된 것으로 생각된다. 고위험 임부의 경우 태아에 대한 걱정과 입원으로 인한 가족과의 분리, 경제적인 부담감 등으로 인해 자신의 상황을 위기로 인식하여 정서적 피로와 우울감을 느낄 수 있어[47] 자존감이 저하될 수 있을 것이다. 따라서 이들에 대한 입원 중의 피로, 우울감에 대한 중재가 필요하며, 여성의 자존감 향상을 위해 집단 독서치료 등이 도움이 되었다는 연구[48]를 통해 볼 때, 고위험 임부의 자존감을 높이기 위해 독서 활동 등을 고려하는 것도 바람직하다.

다소 높은 수준으로 확인된 사회적 지지(평균 10.61점)는 정상 임부를 대상으로 한 선행 연구[40]에서의 평균 98.58점보다 높은 수준을 보였다. 이는 고위험 임신을 진단받은 임부는 불안과 공포, 타인의 비난, 죽음에 대한 두려움, 조산 및 기형에 대한 두려움 등을 경험하지만 의료인의 보살핌과 고위험 임신 질환에 대한 상세한 지침 제공, 대화 등을 통해 자신의 불안의 원인을 명확히 하고 긴장감을 내려놓을 수 있었다는 연구를 고려할 때[49] 본 연구의 대상자가 입원 중에 다양한 지원을 받은 것으로 해석된다. 그러나 COVID-19의 유행 지속으로 입원 상태에 있는 고위험 임부는 면회가 제한되면서 배우자나 가족의 지지에 변화가 발생한 측면도 있으므로, 향후 연구는 입원기간에 따른 사회적 지지 정도를 확인해 볼 필요가 있다.

본 연구에서 고위험 임부의 일반적 특성과 산과적 특성에 따른 모아애착의 차이는 결혼 만족도, 건강상태, 계획 임신 여부에 따라 유의한 차이를 보였다. 먼저 결혼 만족도에 따른 모아애착을 살펴보면, 결혼에 대해 만족하는 경우가 그렇지 않은 경우보다 모아애착이 더 높았다. 이러한 결과는 정상 임부를 대상으로 모아애착 영향요인을 파악한 연구에서 결혼 적응도가 높을수록 모아애착이 높고[20], 배우자와의 상호작용을 통해 좋은 결혼 관계를 유지할 때 결혼 생활의 만족감과 안정감을 느껴 임신을 긍정적으로 받아들이고 모아애착도 높아졌다는 연구 결과[50]와도 일치하는 결과이다. 따라서 모아애착을 높이기 위해서는 부부의 결혼 만족도를 향상할 수 있는 방법을 모색할 필요가 있다고 하겠다.

건강상태에 따른 모아애착은 임부의 건강상태가 보통 수준 이상일 때 높게 나타났다. 이는 정상 임부를 대상으로 모아애착 영향요인을 분석한 연구[51]에서 현재 건강상태가 높다고 인식한 임부의 모아애착이 높았던 것을 통해서도 확인할 수 있다. 고위험 임부의 건강상태는 모아애착 형성을 방해하는 요인이 될 수 있으므로[52] 고위험 임부를 조기 발견하기 위한 예방적인 검사와 지속적인 관리가 필요할 것이다.

계획 임신 여부에 따른 모아애착은 계획 임신을 한 경우가 그렇지 않은 경우보다 점수가 더 높았다. 이는 임산부 교육에 참여한 임부를 대상으로 시행한 연구[12]와 조기진통 임부를 대상으로 시행한 연구[35]에서 계획된 임신이 계획되지 않은 임신보다 더 높은 모아애착 점수를 보인 결과를 통해서도 확인할 수 있다. 따라서 가임기 여성과 예비부부를 대상으로 계획 임신과 산전관리의 중요성을 인지시키고 교육할 필요성이 있다.

본 연구에서 고위험 임부의 모아애착과 사회적 지지, 자존감, 태교실천과의 상관관계를 분석한 결과 모두 정적 상관관계를 보였는데, 이는 사회적 지지, 자존감, 태교실천이 높을수록 모아애착이 높아지는 것을 의미한다. 이러한 결과는 정상 임부를 대상으로 한 연구에서 사회적 지지가 증가할수록 모아애착이 증가하였으며[51], 자존감이 증가할수록 모아애착이 함께 증가하였고[20], 유산을 경험한 임부를 대상으로 한 연구에서 사회적 지지, 태교실천이 높아질수록 모아애착이 높아지는 결과를 통해서도[30] 확인할 수 있다. 따라서 입원 중인 고위험 임부의 사회적 지지를 강화하기 위해 임신 과정에 배우자 및 가족 등이 함께 참여하도록 하고, 자존감 증진을 통해 정서적 안정을 도모하며, 입원 중에도 적극적인 태교실천을 할 수 있도록 고위험 임부를 대상으로 접근성과 활용성을 높인 태교실천 프로그램 적용에 대한 지속적인 관심과 제도 마련이 요구된다.

본 연구는 조사 지역이 국한되어 있으며 3차 의료기관이 제외된 점, 입원 기간을 따로 고려하지 않은 점, 고위험 임신 질환별 표본 수에 차이가 있는 점 등의 제한이 있어 확대 해석에 신중을 기해야 한다. 그러나 모아애착과 관련된 대부분의 선행 연구가 일부 고위험 임부만을 대상으로 하고 있으나 본 연구는 15대 고위험 임신질환을 다양하게 포함하였다는 점에서 의의를 가진다. 향후 다양한 지역의 상급 종합병원에 입원한 고위험 임부를 대상으로 입원 기간을 파악하고, 본 연구에서 현실적 여건에 따라 4대 고위험 임신 질환이 제외되었던 것을 고려하여 19대 고위험 임신 질환별 표본 수 확보를 통한 모아애착에 관한 지속적인 반복 연구가 필요하다. 더하여 임상에서는 고위험 임신을 진단받고 입원한 임부들을 대상으로 태교실천과 사회적 지지를 통해 모아애착을 증진할 수 있도록 접근성과 편의성을 고려한 태교실천 프로그램을 제공하고, 상호 정서적 교류가 가능하도록 병원 내 고위험 임부 자조모임 구성 등을 모색할 필요가 있을 것으로 판단된다. 또한 위계적 회귀분석 시 1단계에서 유의하였던 결혼 만족도와 건강상태가 2단계에서는 모아애착에 영향을 미치는 요인으로 나타나지 않았는데, 이는 2단계에서 투입된 태교실천, 자존감, 사회적 지지와 1단계에서 유의했던 결혼 만족도, 건강상태 간의 매개효과[53]의 가능성이 제기된다. 이는 향후 결혼만족도, 건강상태와 모아애착의 관계에서 태교실천, 자존감, 사회적 지지의 매개효과를 검정하는 연구를 통해 확인해 볼 필요가 있다.

결론적으로, 입원 중인 20주 이후 고위험 임부의 모아애착에 영향을 미치는 요인은 태교실천과 사회적 지지로 확인되었으며 이는 모아애착에 53%의 설명력이 있었다. 고위험 임부를 돌보는 간호사는 입원 중인 고위험 임부의 태교실천과 사회적 지지를 높일 수 있는 중재 역할을 할 수 있으며, 접근성을 높인 입원 중 간호중재 개발이 필요하다.

## Figures and Tables

**Table 1.** Differences in maternal-fetal attachment according to general and obstetric characteristics (N=226)

Characteristic	Categories	n (%)	Maternal-fetal attachment		
			Mean±SD	t/F	*p* ^†^
Age (year)	<35	127 (56.2)	74.87±15.55	0.26	.793
	≥35	99 (43.8)	74.34±13.90		
Education	≤High school	35 (15.5)	70.09±17.99	2.09	.126
	University	166 (73.5)	75.67±14.33		
	≥Graduate school	25 (11.0)	74.12±12.26		
Religion	Yes	82 (36.3)	73.60±14.74	–0.80	.427
	No	144 (63.7)	75.23±14.88		
Employment	Yes	144 (63.7)	73.83±14.57	–1.08	.281
	No	82 (36.3)	76.05±15.24		
Marriage satisfaction	Satisfied^a^	183 (81.0)	77.98±12.48	31.78	<.001
	Moderate^b^	38 (16.8)	61.16±16.52	(a>b,c)	
	Unsatisfied^c^	5 (2.2)	54.60±2.19		
Physical condition	Good^a^	52 (23.0)	79.92±11.88	12.61	<.001
	Moderate^b^	136 (60.2)	75.30±14.85	(a,b>c)	
	Poor^c^	38 (16.8)	65.03±14.19		
Gestational age (week)	<29	78 (34.5)	72.78±16.77	–1.37	.172
	≥29	148 (65.5)	75.61±13.64		
Planned pregnancy	Yes	146 (64.6)	76.90±13.44	2.98	.003
	No	80 (35.4)	70.51±16.36		
Method of pregnancy	Naturally	159 (70.4)	75.38±14.80	1.17	.244
	Infertility procedures	67 (29.6)	72.87±14.84		
Number of pregnancies	First	144 (63.7)	74.18±15.09	–0.61	.541
	≥Second	82 (36.3)	75.44±14.40		

† Scheffé test.

**Table 2.** Scores for maternal-fetal attachment, *taegyo* practices, self-esteem, and social support (N=226)

Variable	Mean±SD	Possible range	Data range
Maternal-fetal attachment	74.64±14.82	24–96	34–96
Practice of *taegyo*	136.47±15.18	37–185	97–178
Self-esteem	35.50±5.90	10–50	21–50
Social support	102.61±22.94	25–125	42–125

**Table 3.** Correlations among maternal-fetal attachment, *taegyo* practices, self-esteem, and social support attachment (N=226)

Variable	r (*p*)		
	Maternal-fetal attachment	Practice of *taegyo*	Self-esteem
Maternal-fetal attachment	1		
Practice of *taegyo*	.70 (<.001)	1	
Self-esteem	.53 (<.001)	.58 (<.001)	1
Social support	.60 (<.001)	.60 (<.001)	.69 (<.001)

**Table 4.** Factors affecting maternal-fetal attachment (N=226)

Variable	Categories	Model 1				Model 2			
		B	β	SE	T (*p*)	B	β	SE	t (*p*)
(Constant)		3.24		.09	34.51 (<.001)	–0.44		.32	–1.35 (.179)
Marriage satisfaction^†^	Moderate	–0.59	–.36	.10	–5.68 (<.001)	–0.11	–.07	.10	–1.15 (.253)
	Unsatisfied	–0.73	–.17	.26	–2.83 (.005)	–0.15	–.04	.21	–.70 (.487)
Physical condition^†^	Moderate	–0.07	–.06	.09	–.80 (.425)	0.01	–.01	.07	.12 (.908)
	Poor	–0.31	–.19	.13	–2.43 (.016)	–0.04	–.03	.10	–.43 (.669)
Planned pregnancy^†^	Yes	0.13	.10	.08	1.63 (.104)	0.06	.05	.06	1.04 (.301)
Practice of *taegyo*						0.76	.50	.09	8.32 (<.001)
Self-esteem						0.08	.08	.07	1.15 (.252)
Social support						0.11	.17	.05	2.18 (.030)
		F (*p*)	14.90 (<.001)				46.28 (<.001)		
		R^2^	.25				.54		
		Adjusted R^2^	.24				.53		
		△R^2^ (*p*)					.29 (<.001)		

† The reference groups were marriage satisfaction (satisfied), physical condition (good), and planned pregnancy (no).
